# MDM2-dependent Sirt1 degradation is a prerequisite for Sirt6-mediated cell death in head and neck cancers

**DOI:** 10.1038/s12276-021-00578-y

**Published:** 2021-03-16

**Authors:** Jung Je Park, Young-Sool Hah, Somi Ryu, So Young Cheon, Seong Jun Won, Jong Sil Lee, Jeong Seok Hwa, Ji Hyun Seo, Hyo Won Chang, Seong Who Kim, Sang Yoon Kim

**Affiliations:** 1 Department of Otolaryngology, Institute of Health Sciences, College of Medicine, Gyeongsang National University, Jinju, South Korea; 2 Biomedical Research Institute, Gyeongsang National University Hospital, Jinju, Republic of Korea; 3 Department of Pathology, Urology and Pediatrics, Gyeongsang National University, Jinju, Korea; 4 Department of Otolaryngology & Department of Biochemistry and Molecular Biology, Asan Medical Center, College of Medicine, University of Ulsan, Seoul, Korea; 5 Department of Biochemistry and Molecular Biology, Asan Medical Center, University of Ulsan College of Medicine, Seoul, Republic of Korea

**Keywords:** Oral cancer, Oral cancer

## Abstract

Sirt6 is involved in multiple biological processes, including aging, metabolism, and tumor suppression. Sirt1, another member of the sirtuin family, functionally overlaps with Sirt6, but its role in tumorigenesis is controversial. In this study, we focused on cell death in association with Sirt6/Sirt1 and reactive oxygen species (ROS) in head and neck squamous cell carcinomas (HNSCCs). Sirt6 induced cell death, as widely reported, but Sirt1 contributed to cell death only when it was suppressed by Sirt6 via regulation of MDM2. Sirt6 and Sirt6-mediated suppression of Sirt1 upregulated ROS, which further led to HNSCC cell death. These results provide insight into the molecular roles of Sirt6 and Sirt1 in tumorigenesis and could therefore contribute to the development of novel strategies to treat HNSCC.

## Cancer: A trio of proteins to tackle tumors

New understanding of the interactions between three proteins sheds light on their role in either promoting or restricting the development of tumors called squamous cell carcinomas, which account for over 90% of all cancers in the head and neck. Researchers in South Korea led by Sang Yoon Kim and Seong Who Kim at the University of Ulsan, Seoul, investigated the role of the proteins Sirt6, Sirt1 and MDM2 in controlling the death of cancer cells caused by chemicals called reactive oxygen species (ROS). The effects of Sirt6 and Sirt1 combine to regulate ROS-induced cancer cell death. Sirt6 controls the activity of MDM2, stimulating ROS production. Sirt6 also influences MDM2 to suppress Sirt1 activity, thereby also promoting cancer cell death. Drugs affecting these three proteins could offer new approaches to anti-cancer therapy.

## Introduction

Head and neck cancer is one of the most common types of cancer, and squamous cell carcinoma accounts for over 90% of all head and neck malignancies^1,2^. In general, therapeutic approaches to cure these malignancies involve surgery combined with radiation therapy or concurrent chemoradiotherapy; however, highly effective treatments for head and neck squamous cell carcinomas (HNSCCs) have not been identified^3,4^. Because HNSCCs are aggressive, various proteins and signaling pathways are considered potential targets of novel curative therapies^3,4^.

Sirtuins are implicated in a wide range of cellular and systemic processes, including energy metabolism, stress responses, and pathological pathways, as well as malignancy^5,6^. Sirt6 is involved in the aging process, including telomere maintenance and DNA repair^7,8^. Sirt6 plays essential biological roles, as shown by the observation that Sirt6 knockout mice have systemic metabolic defects and a shortened lifespan^9^. Moreover, several studies have revealed that Sirt6 is involved in tumor suppression^10,11^. Interestingly, another member of the sirtuin family, Sirt1, overlaps functionally with Sirt6^10^; however, it remains unclear whether Sirt1, a nicotinamide adenine dinucleotide (NAD+)-dependent deacetylase, is a pro- or antitumorigenic molecule^12^. Several lines of evidence suggest that its role is tumor type-specific. This molecule is likely to be protumorigenic in lung, breast, gastric, colon, liver, pancreatic, ovarian, cervical, prostate, and skin cancers^13^, whereas in head and neck cancers, the roles of Sirt1 remain ambiguous, with some studies reporting Sirt1 upregulation and others reporting downregulation^14,15^. Given that many molecules are involved in Sirt1-related mechanisms, understanding the specific functions of Sirt1 is essential for understanding its dual roles.

Various molecules and signaling pathways, including the Forkhead-box transcription factor family, nuclear factor kappa B (NF-κB), and the tumor suppressor p53, are regulated by Sirt6 and Sirt1^16,17^. In particular, the generation of reactive oxygen species (ROS) and oxidative stress signaling regulated by Sirt6 and Sirt1 are key processes in tumorigenesis^18^. When Sirt1 expression is suppressed by Sirt6 via regulation of MDM2 (an E3 ubiquitin–protein ligase), ROS production increases^19,20^. Upregulation of ROS leads to cell death through oxidation of DNA, proteins and many cellular components^20^. In this study, we found that ROS-induced cell death of head and neck cancer cells is initially regulated by Sirt6 and Sirt1. Further elucidation of the Sirt6- and Sirt1-mediated tumorigenic signaling pathways could lead to successful development of therapeutics for HNSCC.

## Materials and methods

### Patients and tissue samples

The study was approved by the Institutional Review Board of the Asan Medical Center (No. 2013-0770). Tissue samples were obtained from patients with stage I–IV tongue cancer who had been diagnosed with SCC and underwent surgery (with or without radiation and chemotherapy) at Asan Medical Center between 2001 and 2010. The medical records of each patient were reviewed; factors recorded included sex, age, TNM stage, pathologic report on resected specimens, treatment modality, recurrence, and death. Each pathologic report included tumor size, tumor differentiation, and margin status of the specimen. TNM stage was determined according to the 2010 AJCC Cancer Staging Manual, Seventh Edition.

### Immunohistochemistry (IHC) for Sirt6 and evaluation of IHC reactions

Formalin-fixed, paraffin-embedded tissue samples from patients with tongue cancer who underwent surgery were subjected to IHC. Tissue microarray blocks were cut into 4 µm slices, which were deparaffinized and rehydrated. The slides were incubated in 3% hydrogen peroxide for 10 min to block endogenous peroxidase activity and then heated for 20 min in 10 mM citrate buffer (pH 6.0) in a microwave oven (700 W). The sections were incubated overnight at 4 °C with anti-sirtuin6 antibodies (Abcam, Cambridge, UK). The IHC results were independently examined by two pathologists blinded to the patient data.

### Cell culture

HNSCC cell lines (HN-30, HN-31, UMSCC-1, UMSCC-47, and UMSCC-38) and an immortalized nontumorigenic cell line (HaCaT) were used in this study. All UMSCC lines (University of Michigan Squamous Cell Carcinomas) were kindly provided by Dr. T. Carey (University of Michigan, Ann Arbor, USA). These cell lines were maintained in Dulbecco’s modified Eagle’s medium (DMEM) (Thermo Fisher Scientific, Waltham, MA, USA) supplemented with 10% fetal bovine serum (Thermo Fisher Scientific) and 1% penicillin/streptomycin (Thermo Fisher Scientific) and incubated at 37 °C with 5% CO_2_ in a humidified incubator.

### Preparation of adenovirus

Adenovirus encoding Sirt6 (Ad-Sirt6) was created using the ViraPower adenovirus expression system (Invitrogen, Thermo Fisher Scientific, USA). Briefly, cDNA encoding Sirt6 was subcloned into pENTR. After sequence verification, the Sirt6 cDNA was cloned into the pAd/CMV/V5-DEST vector using the Gateway system with LR Clonase (Invitrogen). The verified clone (Ad-Sirt6) was linearized using *Pac*I (New England Biolab) and then transfected into 293A cells with Lipofectamine 3000 (Invitrogen). The virus was prepared and amplified with the ViraPower adenoviral expression system (Invitrogen), and viral titers were determined by plaque-forming assays with serial dilution. Aliquots of viral suspension were used to infect HaCaT and HNSCC cell lines. Recombinant replication-defective adenovirus encoding green fluorescent protein (Ad-GFP) and β-galactosidase (Ad-LacZ) was used as a control.

### Flow cytometric analysis

Collected cells were washed twice with cold PBS, fixed with 70% ethanol for 1 h at 4 °C, treated with 1 mg/ml RNase A (Sigma-Aldrich, St. Louis, MO, USA) and then stained with 50 μg/ml PI (Sigma-Aldrich). Data were acquired on a Cytomics FC500 Flow Cytometer equipped with two laser sources (Beckman-Coulter). The results were analyzed using CXP Software (Beckman-Coulter).

### In vivo xenograft mouse model

All animal experiments were approved by the Institutional Animal Care and Use Committee (IACUC) of Gyeongsang National University and conducted according to the National Research Council Guidelines. A cell suspension (5 × 10^6^ cells/mouse) of HN31 was injected subcutaneously into 6-week-old male nude mice (athymic nude mice; Harlan, Indianapolis, IN, USA). Nine days after inoculation of the cells, animals with xenografts that were 0.6–0.7 cm in diameter were treated with intratumoral injections of Ad-GFP or Ad-Sirt6. The tumor diameters were measured with digital calipers on days 3, 7, 10, and 14, and the tumor volume was determined using the modified ellipsoidal formula (tumor volume = 1/2[length × width^2^]). The tumor size of HN31 flank xenografts was determined for 32 days after cell inoculation, and xenografts were excised and weighed on day 32.

### ROS measurement

Intracellular generation of ROS was measured using 2′,7′-dichlorodihydrofluorescein diacetate (DCF-DA; Molecular Probes, Eugene, OR, USA). Cells were stained with 5 μM DCF-DA in serum-free medium for 15 min and removed from the plate with trypsin–EDTA (Gibco/BRL). The fluorescence intensity of the cells was measured by flow cytometry with an excitation wavelength of 480 nm and an emission wavelength of 525 nm. Data were analyzed using CXP software.

### Short-hairpin RNA (shRNA)-mediated silencing of Sirt6 and MDM2

For shRNA-mediated depletion of Sirt6 and MDM2, glycerol stocks of bacteria containing Sirt6- or MDM2-targeting shRNA plasmid DNA (MISSION shRNA), as well as a nontargeting control plasmid DNA (SHC002), were purchased from Sigma-Aldrich. Lentivirus particles were used to deliver and express shRNAs to knock down human Sirt6 and MDM2, and a nontarget scrambled shRNA was used as a control. Lentivirus particles were generated by cotransfection of a targeting set of shRNA plasmids (Sirt6 and MDM2) or nontargeting control shRNA plasmid along with MISSION Lentiviral Packaging Mix (SHP001; Sigma-Aldrich) into 293FT cells (Thermo Fisher Scientific) using Lipofectamine 3000 (Life Technologies, Germany). Cell culture supernatants containing lentivirus particles were collected at 24 and 48 h post transfection, filtered, and used to infect HNSCC cell lines. The efficiencies of Sirt6 and MDM2 knockdown were evaluated by western blotting of whole-cell extracts.

### Western blot analysis

Total proteins were extracted from HNSCC cells with radioimmunoprecipitation assay lysis buffer supplemented with protease inhibitor cocktail (Calbiochem, San Diego, CA, USA). Protein concentrations were determined using the BCA protein assay kit (Pierce, Rockford, IL, USA). Total protein lysates (30 μg) were separated by sodium dodecyl sulfate-polyacrylamide gel electrophoresis (SDS-PAGE) and transferred to nitrocellulose membranes (Millipore, Bedford, MA, USA). Protein bands were visualized with enhanced chemiluminescence detection reagent (Pierce) and imaged using the ChemiDoc Touch Imaging System (Bio-Rad, Hercules, CA, USA).

### Preparation of nuclear and cytosolic extracts

Nuclear and cytoplasmic fractions were prepared from cells using NE-PER Reagent (Pierce). Briefly, cells were harvested in trypsin-EDTA (MediaTek) and centrifuged at 500*g* for 3 min. Cell pellets were resuspended in cytoplasmic extraction reagents (CERI and CERII), vortexed at high speed for 15 s, and centrifuged at 16,000*g* for 5 min at 4 °C. Cytoplasmic protein was recovered from the supernatant. The pellet was resuspended in nuclear extraction buffer by intermittent high-speed vortexing over 40 min, and the sample was centrifuged at 16,000*g* for 10 min at 4 °C. Nuclear protein was recovered from the supernatant. Protein concentrations of the nuclear and cytoplasmic fractions were determined using the BCA assay. Cytosolic and nuclear proteins or whole-cell lysates were separated by SDS-PAGE in a 10% polyacrylamide gel and transferred to a nitrocellulose membrane (Millipore, Bedford, MA, USA). Membranes were incubated with primary antibodies against lamin A/C (Santa Cruz Biotechnology, Dallas, TX, USA) and α-tubulin (Sigma, St. Louis, MO, USA), followed by incubation with horseradish peroxidase-conjugated anti-rabbit immunoglobulin (Ig) G or anti-mouse IgG (Cell Signaling Technology, Beverly, MA, USA). Antibody binding was detected using an enhanced chemiluminescence detection reagent (Pierce). Images were acquired with the ChemiDoc Touch Imaging System (Bio-Rad).

### Immunoprecipitation

Cells were grown in 100 mm culture dishes and lysed in homogenizing buffer. Lysates were incubated overnight at 4 °C with 10 µl of mouse IgG or rabbit IgG against ubiquitin, MDM2, Sirt6, or acetyl-lysine. After incubation for 1 h with protein A/G magnetic beads (Thermo Fisher Scientific), immunoprecipitates were resuspended in Laemmli buffer, subjected to 10% polyacrylamide gel electrophoresis, and transferred to nitrocellulose membranes. Sirt1, Sirt6, and MDM2 were specifically detected by western blots.

### Statistics

Disease-free survival (DFS) and overall survival (OS) were determined by the Kaplan–Meier method and compared using log-rank tests. In survival analyses, the follow-up duration was defined as the time from date of diagnosis to the date of recurrence or death to a maximum of 10 years. Factors significantly predictive of DFS and OS were determined by univariate and multivariate analyses using Cox’s proportional hazards regression model and are reported as hazard ratios and 95% confidence intervals.

GraphPad Prism (Version 5.01; GraphPad Software, San Diego, CA, USA) was used for statistical analysis. Data are shown as the mean ± SD. Student’s *t* test and one-way ANOVA were conducted to analyze the differences between and among groups, respectively. A *p* value < 0.05 was considered significant.

## Results

**Fig. 1:**
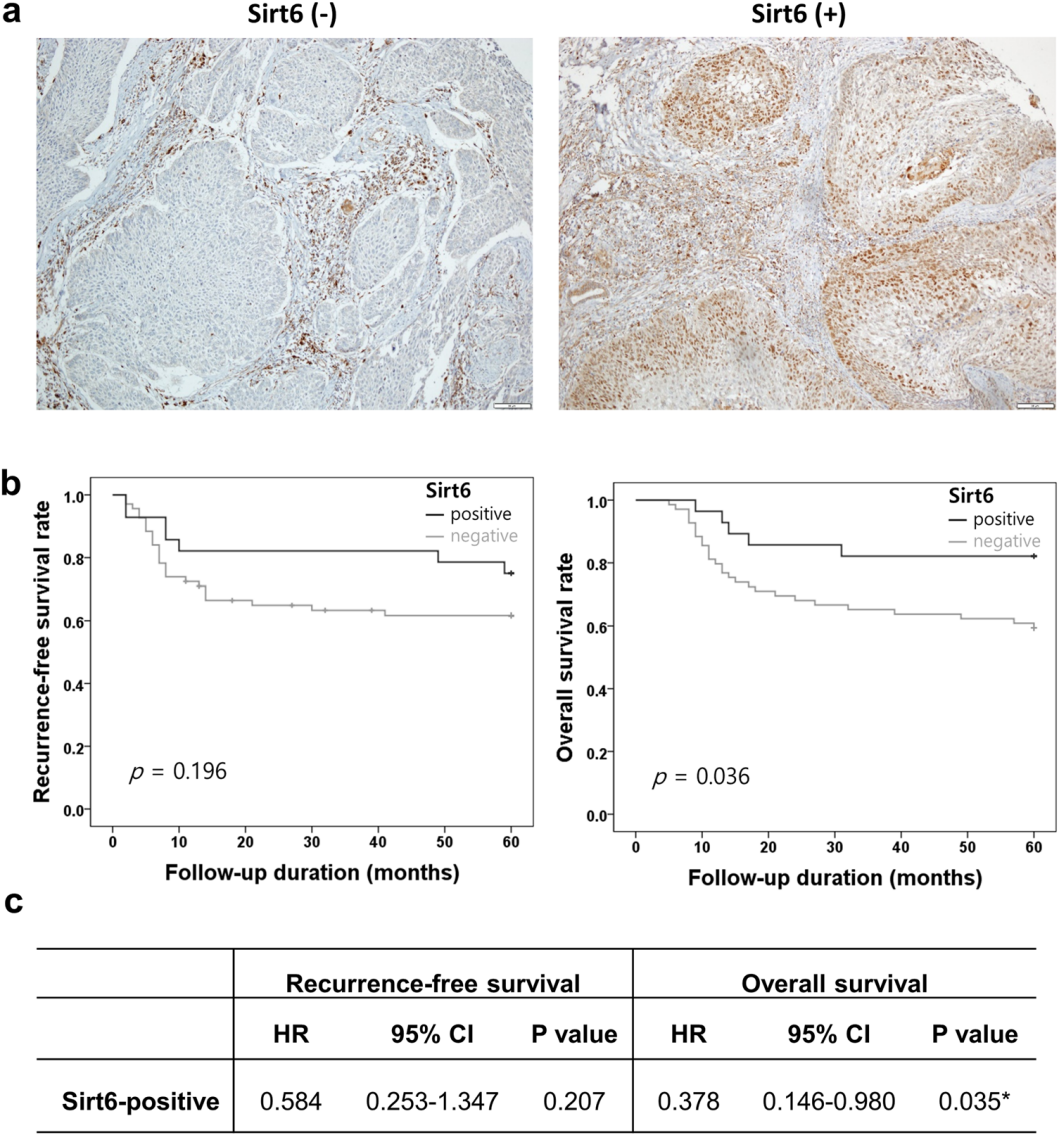
Clinical significance of Sirt6 expression in HNSCC. Sirt6 is downregulated in HNSCC. **a** Representative IHC staining of HNSCC tissues with different degrees of Sirt6 expression. Number of positive cells: (−) <10%; (+) >10%. **b** Kaplan–Meier curve of HNSCC patients with positive or negative expression of Sirt6 (*n* = 97; *p < 0.05). **c** Correlation between Sirt6 expression and survival in 96 tongue cancer patients (*p < 0.05). The Sirt6-positive tongue cancer patients had a higher overall survival rate than the Sirt6-negative patients, but Sirt6 expression did not affect recurrence-free survival.

A comparison of the survival rates of the Sirt6-positive and Sirt6-negative HNSCC cancer patients revealed that the Sirt6-positive patients had a higher OS rate (*p* = 0.036) over 5 years than the Sirt6-negative patients, although the recurrence-free survival rate did not significantly differ between the groups (*p* = 0.196) (Fig. 1a, b). Among 97 tongue SCC patients, the Sirt6-positive patients exhibited a better prognosis than the Sirt6-negative patients. The Sirt6-positive tongue SCC patients had a higher OS rate (*p* = 0.036) over 5 years than the Sirt6-negative patients, although the recurrence-free survival rate did not significantly differ between the groups (*p* = 0.207) (Fig. 1c). These results indicate that Sirt6 has important biological functions in HNSCC tumorigenesis.

**Fig. 2:**
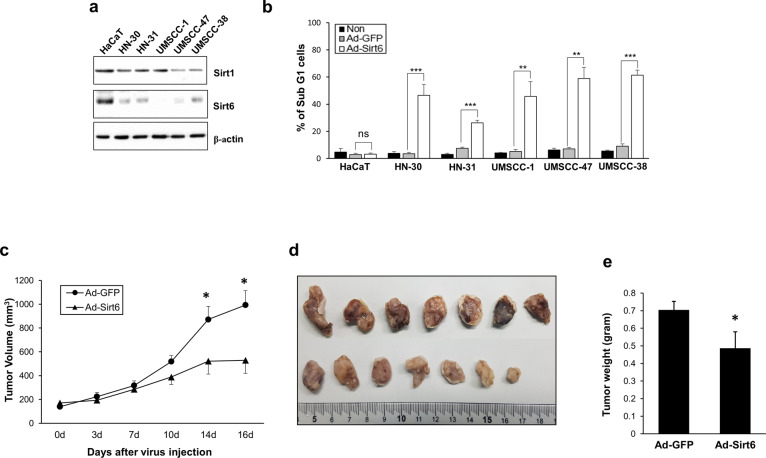
Sirt6 has negative effects on survival of HNSCC cells. **a** Sirt6 expression is downregulated in human HNSCC cell lines. Western blotting was performed to determine Sirt and Sirt6 expression in HNSCC cell lines and the immortalized human keratinocyte cell line HaCaT. **b** Sirt6 overexpression was selectively cytotoxic in multiple HNSCC cell lines but not in HaCaT cells. **c**–**e** Effect of Sirt6 on HNSCC growth in a xenograft mouse model. **c** Sirt6 significantly inhibited tumor growth in a xenograft mouse model. Average tumor volumes in the vehicle-treated control mice and the Sirt6-treated mice were plotted over 16 days after tumor cell injection. The tumor volume was measured by calipers. Asterisks indicate a significant difference in tumor size (*p < 0.05). Data are presented as the mean ± SEM (standard error of the mean) and were analyzed using Student’s *t* test. **d** Tumor images represent excised tumors from each group, comparing the Sirt6-expression plasmid group to the control plasmid group. **e** Tumor weights compared between the Sirt6-expression plasmid group and the control plasmid group. Tumor growth was substantially inhibited in the Sirt6-treated group, whereas tumors in the control group continued to grow (*p < 0.05).

Next, we assessed the Sirt1 and Sirt6 expression levels in multiple HNSCC cell lines (HN-30, HN-31, UMSCC-1, UMSCC-47, and UMSCC-38). Sirt6 was expressed at lower levels in the HNSCC cells than in the control HaCaT cells, whereas the Sirt1 expression levels did not significantly differ between the two cell types (Fig. 2a). The percentage of sub-G1 cells (indicating cell death) following treatment with Ad-Sirt6 was substantially lower in all HNSCC cell lines than the control cell line (Fig. 2b). In a xenograft mouse model using HN31, which does not express Sirt6, intratumoral injection of Ad-Sirt6 significantly decreased tumor growth, volume, and weight (Fig. 2c–e). These data indicated that Sirt6 has negative effects on the survival of HNSCC cells.

**Fig. 3:**
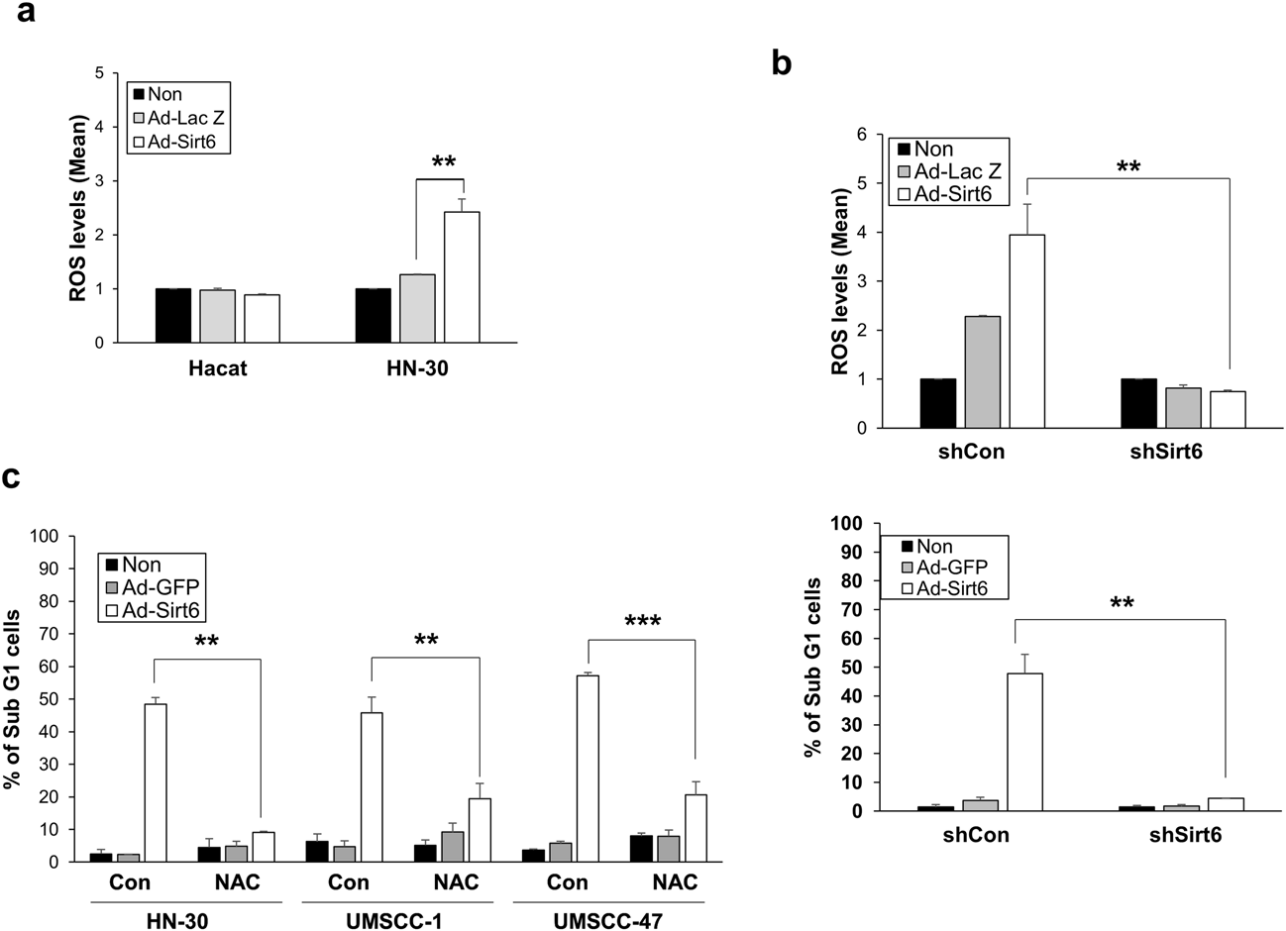
Sirt6 inhibits cell survival and promotes cell death by modulating reactive oxygen species (ROS) levels in HNSCC cells. **a** HaCaT cells were seeded in 96-well plates, and then, the ROS levels were measured after 48 h using the fluorogenic marker carboxy-2,7-dichlorodihydrofluorescein diacetate (H2DCFDA). The ROS levels were unchanged in the Sirt6-overexpressing HaCaT cells but substantially increased in the Sirt6-overexpressing HNSCC cells (**p < 0.01). **b** The ROS levels were downregulated following shSirt6-mediated knockdown of Sirt6, and the percentage of sub-G1 cells was also reduced (**p < 0.01). **c** For further confirmation that Sirt6 functions in HNSCC cells by modulating ROS levels, the ROS scavenger NAC was used. The addition of NAC abrogated the effect of Sirt6 on HNSCC cells, leading to elevated ROS levels, increased cell viability and decreased cell death (**p < 0.01, ***p < 0.001).

In cancer-associated pathways, ROS are crucial for modulating Sirt6 to inhibit cell survival and promote cell death. We compared the ROS levels after transfection of Sirt6 into HaCaT and HN-30 cells. The ROS levels were unchanged in the HaCaT cells but were elevated in the HN-30 cells, indicating that Sirt6 increases the ROS levels in HNSCC cells (Fig. 3a). The ROS levels were reduced when Sirt6 expression was inhibited with shSirt6, and the percentage of sub-G1 cells was also decreased by Sirt6 inhibition (Fig. 3b). The addition of NAC, an ROS scavenger, decreased the effect of Sirt6-induced cell death in various HNSCC cells (Fig. 3c). These data indicated that Sirt6 induces cell death by modulating ROS levels.

**Fig. 4:**
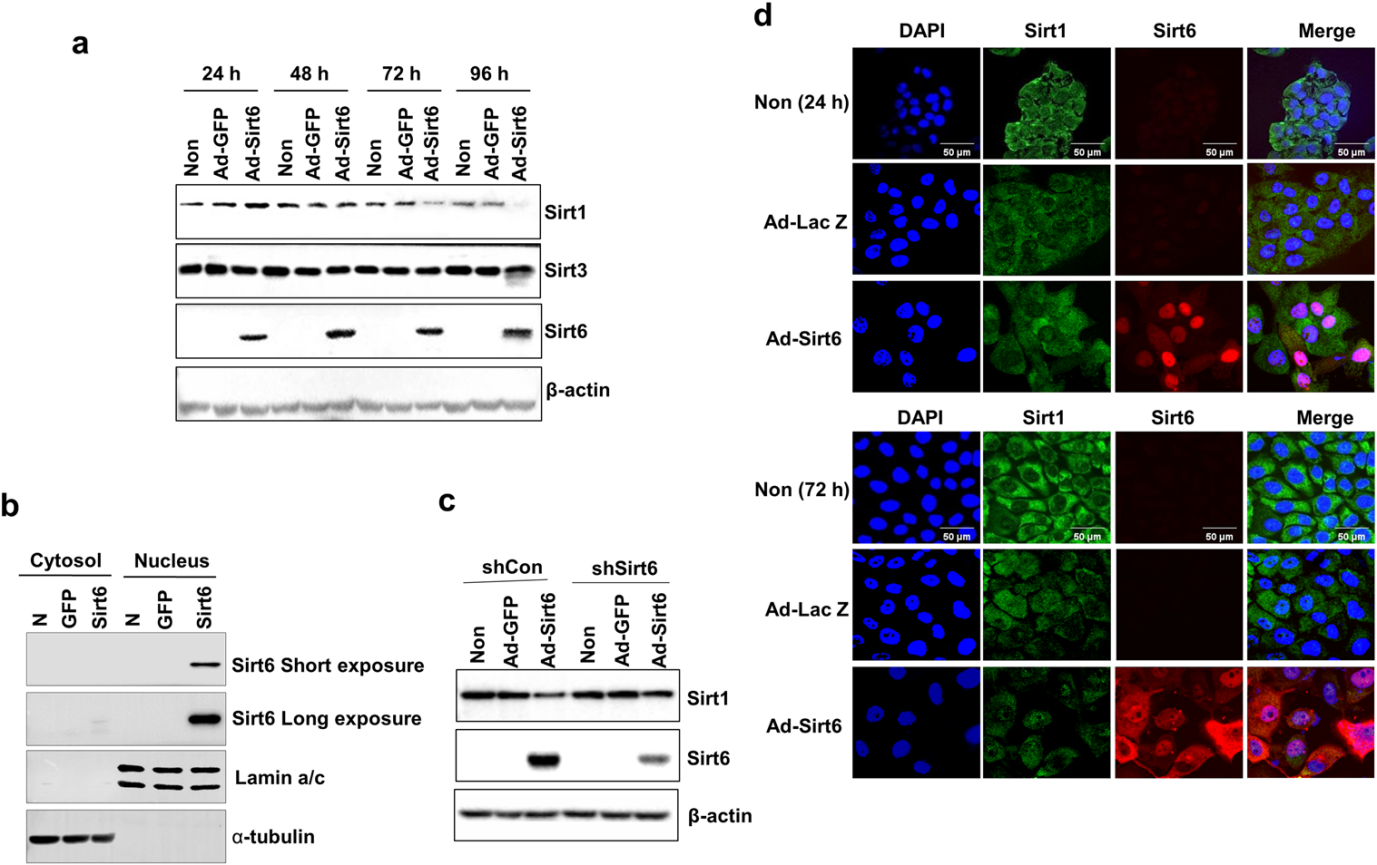
Sirt1 and Sirt6 play interrelated roles in HNSCC cell survival. **a** The expression of Sirt1 was reduced, whereas the expression of Sirt6 was increased, in a time-dependent manner when Sirt6 was overexpressed from Ad-Sirt6. Sirt3 expression was not affected by Sirt6 overexpression. **b** Sirt6 expression was detected in the nucleus but not the cytoplasm. **c** Sirt6 expression was inhibited by shSirt6, whereas Sirt1 expression was not affected. **d** Fluorescence images of HNSCC cells showing Sirt1 and Sirt6 levels 24 and 72 h after Sirt6 overexpression. Sirt1 was downregulated and Sirt6 was upregulated by Sirt6 overexpression from Ad-Sirt6, and Sirt6 seemed to move from the nucleus (24 h) to the cytoplasm (72 h).

As shown in Fig. 4a, Sirt1 expression was decreased in a time-dependent manner by Sirt6, whereas Sirt3 expression was not affected (Fig. 4a). Sirt6 was mainly localized in the nucleus rather than the cytoplasm (Fig. 4b). Sirt1 expression was inhibited when Sirt6 was overexpressed and recovered when Sirt6 expression was knocked down by shSirt6 (Fig. 4c). Fluorescence images of HNSCC cells revealed that Sirt6 moved from the nucleus at 24 h to the cytoplasm at 72 h (Fig. 4d). Fluorescence imaging demonstrated that Sirt1 was downregulated by Sirt6 overexpression from Ad-Sirt6 at both time points (24 and 72 h) (Fig. 4d). Together, these results indicated that Sirt1 and Sirt6 play interrelated roles in HNSCC cell survival.

**Fig. 5:**
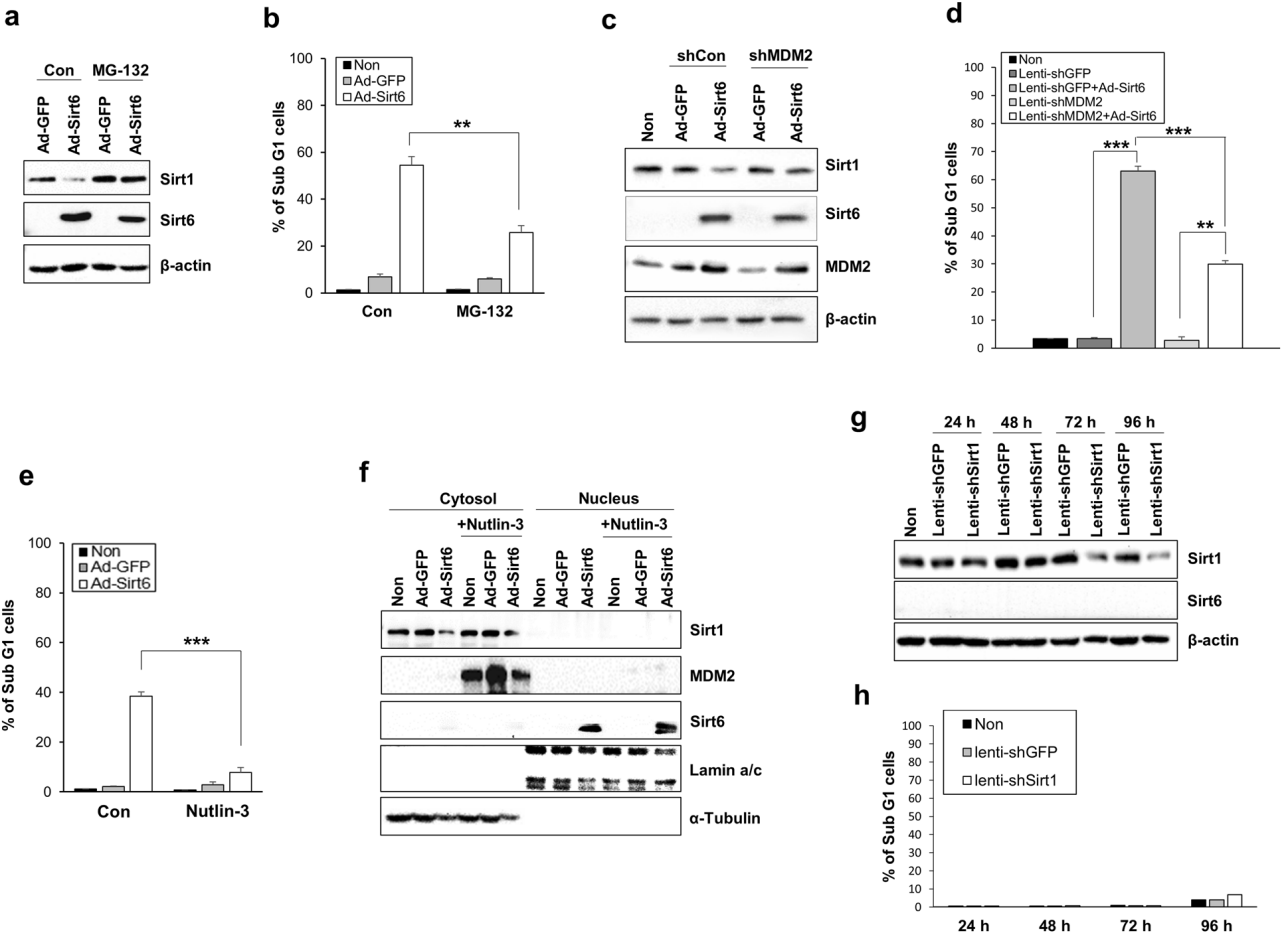
HNSCC cell death is induced when Sirt1 is suppressed by Sirt6. **a** In the Sirt6-overexpressing HNSCC cells in the presence of a 26S proteasome inhibitor (MG-132), Sirt1 expression was increased and Sirt6 expression was decreased. b MG-132 treatment decreased the percentage of sub-G1 cells, representing a reduction in apoptotic activity, in the Sirt6-overexpressing HNSCC cells (**p < 0.01). **c** The Sirt6-overexpressing cells had elevated MDM2 levels and reduced Sirt1 levels, and MDM2 knockdown using shMDM2 downregulated Sirt6 and upregulated Sirt1. **d** The percentage of sub-G1 cells was significantly increased by Sirt6 overexpression and decreased by MDM2 knockdown (**p < 0.01, ***p < 0.001). **e** Treatment with the MDM2 antagonist Nutlin-3 decreased the frequency of cell death in the Sirt6-overexpressing HNSCC cells (***p < 0.001). f MDM2 expression was elevated upon Nutlin-3 treatment because the drug targets the p53–MDM2 interaction. Sirt1, which is mainly localized in the cytosol, was not downregulated by MDM2 inhibition using Nutlin-3; by contrast, Sirt6, which translocates from the nucleus to the cytosol, was upregulated. **g** Sirt6 expression was not altered by Sirt1 knockdown using shSirt1. **h** Sirt1 inhibition did not significantly affect the percentage of sub-G1 cells.

Because the proteasome is responsible for the removal of oxidatively damaged proteins from the cytosol and nucleus, we investigated whether the proteasome-mediated protein degradation pathway is linked to Sirt1 cleavage in HNSCC cells. Sirt1 expression was elevated, whereas Sirt6 expression was reduced, when the Sirt6-overexpressing HNSCC cells were treated with the 26S proteasome inhibitor MG-132 (Fig. 5a). MG-132 treatment suppressed Sirt6-induced cell death (Fig. 5b). In addition, we used shMDM2 to explore the relationship among MDM2, Sirt1, and Sirt6. MDM2 knockdown downregulated Sirt6 but upregulated Sirt1 (Fig. 5c). The percentage of sub-G1 cells was significantly increased upon Sirt6 overexpression, whereas cell death was diminished when MDM2 was knocked down (Fig. 5d). Sirt6-induced cell death was substantially suppressed upon inhibition of MDM2, indicating that MDM2 plays an essential role in Sirt6-related cancer cell death (Fig. 5d). Treatment with the MDM2 antagonist Nutlin-3 decreased the frequency of cell death in the Sirt6-overexpressing HNSCC cells (Fig. 5e). Nutlin-3 inhibits MDM2 by targeting the p53–MDM2 interaction; consistent with this finding, MDM2 expression increased following Nutlin-3 treatment. When MDM2 was inhibited by Nutlin-3, Sirt1 (which is primarily localized in the cytosol) was not downregulated, whereas Sirt6 (which translocates from the nucleus to the cytosol) was upregulated (Fig. 5f). When Sirt1 expression was suppressed by shSirt1, Sirt6 expression (Fig. 5g) and the percentage of sub-G1 cells were unchanged (Fig. 5h). These data indicated that cancer cell death is induced when Sirt1 is suppressed by Sirt6, whereas inhibition of Sirt1 without Sirt6 expression does not significantly affect cell death.

**Fig. 6:**
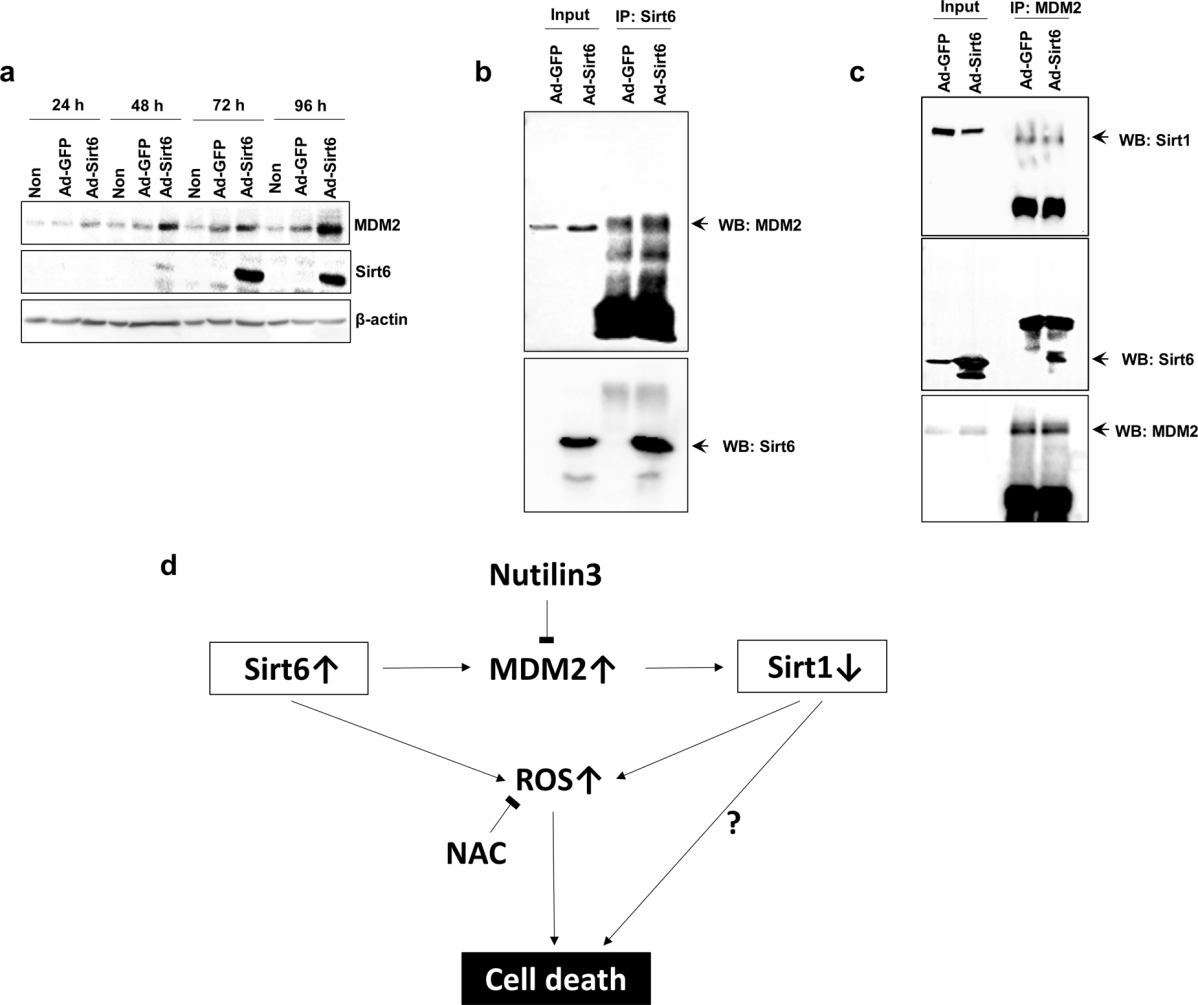
Interaction betwen MDM2 adn Sirt6. **a** The MDM2 level increased in a time-dependent manner when Sirt6 was overexpressed from Ad-Sirt6. **b** MDM2 was upregulated in the presence of Sirt6, as demonstrated by SDS-PAGE. **c** Both Sirt1 and Sirt6 were downregulated in the presence of MDM2. **d** Schematic diagram of Sirt6- and Sirt1-mediated cell death pathways in HNSCC cells.

MDM2 expression was upregulated in a time-dependent manner when Sirt6 was overexpressed from Ad-Sirt6 (Fig. 6a). Interestingly, interactions between MDM2 and Sirt6 were increased in the presence of Sirt6, as revealed by a coimmunoprecipitation assays (Fig. 6b), and Sirt1 was downregulated in the presence of MDM2 and Sirt6 (Fig. 6c). The overall roles of Sirt6 and Sirt1 in HNSCC tumorigenesis are shown schematically in Fig. 6d. Sirt6 induces MDM2, which can further downregulate Sirt1 expression; in addition, both Sirt6 upregulation and Sirt1 downregulation induce ROS expression, leading to Sirt6- or Sirt1-induced death of HNSCC cells. Although Sirt1 upregulates ROS expression, leading to cancer cell death, Sirt1 itself does not promote cell death to the same extent.

## Discussion

Because head and neck cancer includes various subpopulations of cells and interactions among multiple genetic pathways, current treatments for HNSCC are not always successful^1^. Although advances in surgery, radiotherapy, and chemotherapy have led to better control of localized cancer cells and OS, many HNSCC patients still suffer from tumor relapse and metastasis associated with treatment failure^3^. Therefore, a better understanding of the molecular mechanisms underlying the tumorigenic process of HNSCC is crucial for the development of novel strategies to treat HNSCC.

Among the various target molecules in HNSCC, Sirt6 is a member of the Sirt family, which has a wide range of biological roles^7^. Multiple studies have reported a correlation between survival rate and Sirt6 level in various cancers; in this study, the Sirt6-positive tongue cancer patients had a higher survival rate than the Sirt6-negative patients. In addition, the tumor volume and weight were significantly lower in an HNSCC-injected xenograft mouse model than in the control. These results strongly suggest that Sirt6 has an antitumor effect and should therefore be considered a new therapeutic target for head and neck cancer.

ROS, a byproduct of oxygen metabolism, promote cell death through oxidation of various biological components^21^. Upregulation of ROS is especially crucial in modulating Sirt6 to inhibit cell survival and promote cell death in both healthy and malignant cells^20^. The anticancer effect of Sirt6 was influenced by upregulation of ROS, as Sirt6 overexpression significantly increased ROS expression, whereas NAC (a scavenger for ROS) decreased the apoptotic activity of Sirt6 in HNSCC cells. Because ROS affect the expression levels of various molecules and sequential cascades that lead to cell death, fine-tuning of ROS signaling is necessary for further development of an effective novel treatment^22^. MDM2, a negative regulator of the p53 tumor suppressor, also plays a key role in the antitumor effect of Sirt6^23^. MDM2 degrades Sirt6 in a proteasome-dependent manner^23^. This molecule plays tumor-related roles in a number of malignancies, including lung cancer and breast cancer, but its effect in HNSCC has not been extensively studied^24^. Here, we showed that MDM2-mediated regulation of Sirt6 decreased the percentage of sub-G1 HNSCC cells (representing cell death).

Although Sirt6 exerts an antitumorigenic effect by regulating ROS and MDM2, it remains unclear whether Sirt1 is pro- or antitumorigenic^12^. Many studies have revealed a protumorigenic effect of Sirt1 in many types of cancer, but its role in head and neck cancer remains controversial^13–15^. Therefore, it is important to understand its dual role in a tumor-specific and pathway-specific manner. As shown in this study, Sirt1 is likely to aggravate malignant development: we observed an increase in ROS expression, leading to an elevated frequency of cancer cell death, when Sirt1 was downregulated by Sirt6. Our findings demonstrate, for the first time, the correlation between Sirt6-mediated Sirt1 and ROS regulation in HNSCC, although a more comprehensive study regarding the specific underlying pathway to explain why Sirt1 downregulation itself is not enough to induce tumor cell death is required in the future.

In summary, we have revealed the molecular mechanisms underlying the tumorigenic effects of Sirt6 and Sirt6-mediated suppression of Sirt1. Our findings confirmed that the antitumorigenic effect of Sirt6 in HNSCC is mediated by the regulation of MDM2 and ROS. However, Sirt1 is protumorigenic only when it is suppressed by MDM2, resulting in upregulation of ROS. These observations will help elucidate the dual role of Sirt1 in various types of cancer, and this new knowledge should facilitate the development of novel strategies for treating HNSCC.
